# The Moderation of Obesity Penalty on Job Market Outcomes by Employment Efforts

**DOI:** 10.3390/ijerph16162974

**Published:** 2019-08-19

**Authors:** Rosemary Ahn, Tae Hyun Kim, Euna Han

**Affiliations:** 1 The Fuqua School of Business, Duke University, Durham, NC 27708, USA; 2 Graduate School of Public Health and Institute of Health Services Research, Yonsei University, Seoul 037252, Korea; 3 College of Pharmacy, Institute of Pharmaceutical Sciences, Yonsei University, Incheon 21983, Korea

**Keywords:** Job efforts, obesity penalty, job performances, young adults

## Abstract

The current study explores the moderation of the relationship between obesity and labor market outcomes by direct employment efforts such as job hunting and job training of young adults. The study used data provided by the Korean Education and Employment Panel, a longitudinal data survey comprising middle and high school students from 2004 to 2015. Two dependent variables were assessed in this study: employment status and wage. The individual-level fixed effects were controlled. Despite having more direct employment efforts of either or both experience in job hunting and job training, compared to normal-weight counterparts, underweight men and overweight and obese women were reported to have a disadvantage in both dependent variables. Underweight men with job training experience were 12.02% less likely to be employed, while overweight and obese men had 6.80 times higher monthly wages when job training experience was accompanied compared to no such experience. For overweight and obese women, compared to that of their normal-weight counterparts, employment probability decreased by 4.78% per week-increase in job hunting, by 2.81% if any experience in job hunting. For underweight women, compared to that of their normal-weight counterparts, employment probability increased by 4.56 times per week-increase in job hunting and by 5.59 times if experience in job hunting, and by 6.96% if experience in job training. The results indicate that employment efforts do not fully moderate the presence of obesity penalty for labor market outcomes on those early in their careers.

## 1. Introduction

Obesity is known to be a major health issue that has become prevalent all over the world. The obesity rate for American adults rose from 22.9% in 1988 to 39.6% in 2016 [1]. The adult obesity rates for 5 of the 50 states of the United States exceeded 35%, and at least 46 states exceeded 25% in 2017 [2]. Likewise, the obesity rate in South Korea rose from 25% in 1998 to 31% in 2014 [3].

Obesity is linked to a series of negative effects. Specifically, previous studies have found obesity to have notable influence in labor market outcomes [4,5]. Body weight penalty, which involves biased judgment on one’s personality and behavior, has been increasingly observed to be present in the labor market [6]. For instance, overweight and obese individuals are less inclined to have a determined mindset, high test scores, and ambition for higher education because of the prejudice they face in their daily lives [7,8]. In addition, characteristics like anxiety, depressive symptoms, and an overall lower mental well-being have been observed in this particular weight category [9]. Weight discrimination not only affects these individuals’ personality traits early in their lives but also facilitates their lack of motivation and drive later on in their careers [10]. Furthermore, obese people are more likely to have higher rates of time preference and less likely to invest in future long-term goals than lower-weight individuals, preventing them from being aware of their potential [11].

Another factor that takes part in prejudice toward overweight and obese individuals relates to health issues and their impact upon employment [12,13,14]. Health issues from obesity lead to an increase in required health care and decrease in work performance, resulting in an increase in company costs of hiring an obese employee, which would potentially decrease wages, compared to those of normal-weight individuals [10,15].

For such reasons, the association between obesity and the labor market has been a widely known issue in previous literature. The majority of the studies have shown that obese individuals face more weight discrimination than normal-weight individuals [6]. While previous studies have consistently researched the relationship between obesity and employment [16], the effects of obesity in a combination with direct employment efforts such as both job training and job hunting on job market outcomes remain less explored [16,17]. Applicants make direct employment efforts with expectations in positive labor market performances like employment, wage growth, job performance, and job upgrades [18,19,20]. Therefore, this paper dives deeper into the relationship between obesity and labor market outcomes by incorporating employment efforts as a moderator. In addition, this paper concentrates on young adults in their twenties, a period in which individuals are generally new to the labor market.

## 2. Materials and Methods

### 2.1. Data and Study Subjects

The study uses data provided by the Korean Education and Employment Panel (KEEP), a longitudinal survey data comprising middle school and high school students gathered from 2004 to 2015. The data provides information on the students’ education background, employment details, and other personal information. KEEP gathered different information from high school and middle school students by sending a different questionnaire to each—one for middle school students focused on school life and one for high school students focused on employment. Since there is no job-related information that can be obtained from the middle school students, the study only focuses on high school students.

**Figure 1:**
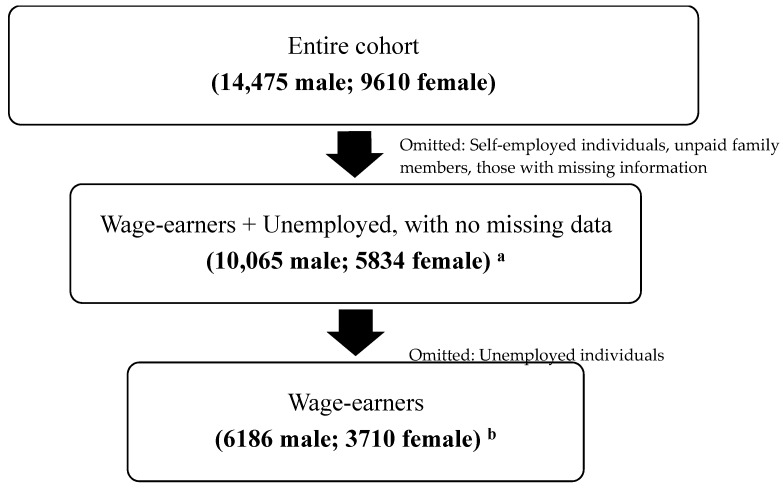
A flowchart demonstrating the obtained final sample used for the study. Note: ^a^ This sample was used to assess the respondents’ current employment status, key independent variables, and covariates, as seen on Table 1. ^b^ This final sample of wage-earners was used to assess the respondents wage levels to analyze the moderating effects of job hunting and job training in the labor market.

From the initial dataset of 9610 female and 14,475 male high school respondents in 2004, this study used data on these high school students from 2004 to 2015 who are either unemployed or have had any experience in being a wage earner. Since this study is designed to examine the respondents’ wage level, those that had experience in having occupations labeled under the “unpaid family member” and/or “self-employed” categories were excluded from the investigation, resulting in a count of 9293 female and 13,624 male individuals, and respondents who had missing data in any of the questions asked (excluding the questions regarding parents’ income) were excluded. Moreover, lagged BMI was used for this study, so respondents without BMI details from previous years were omitted, and observations with lagged BMI of fewer than 10 or over 40 were also excluded. As a result, a total of 10,065 male and 5834 female high school students from 2004 were extracted to analyze their responses for 13 years (from 2004 to 2015) and further conduct this study. Among these respondents, 6186 male and 3710 female students were thus further assessed as the employed sample persons (Figure 1).

### 2.2. Variables

The two dependent variables for this study were employment status and wage. *Employment status* is represented with binary variables. This study focuses on wage earners since unpaid family members and self-employed workers lack thorough information in the KEEP survey. *Wages* were measured on a monthly basis in units of KRW in this dataset. The general conversion rate that could be used as reference for this study was 1000 KRW = 0.82 USD.

The independent variables for this study are the respondents’ lagged BMI, lagged job hunting duration, and lagged number of job training experiences. BMI is calculated by dividing weight in kilograms by height in meters squared, and three BMI classification groups were generated: underweight (BMI under 18.5), normal-weight (BMI between 18.5 and 25), and overweight and obese (BMI of over 25). BMI obtained from the previous year’s survey was used as lagged BMI. Data on job hunting duration were collected in week units and revised to calculate the accumulative sum of weeks spent in job hunting prior to employment. Likewise, job-training experiences in terms of number of experiences were used to create an independent variable that comprises the accumulative sum of job training experiences right until employment.

The following variables were set as covariates as a series of dummy indicators: highest level of educational attainment (high school diploma or less, college diploma or more); college letter grade (A, B, C, D/F); second language experience (any experience in learning any language other than their native language); father’s average monthly income (0–2 million KRW, 2–3 million KRW, 3–5 million KRW, 5+ million KRW); mother’s average monthly income (0–2 million KRW, 2–3 million KRW, 3–5 million KRW, 5+ million KRW); self-reported health status (healthy, average, unhealthy); frequency of breakfast intake (often, sometimes, never); and any perceived physical discrimination. Average hours of sleep per day was measured as a linear variable. Perceived awareness of self was divided into six different traits: aptitude, interests, value, decisiveness, perseverance, and image/reputation—all of which are represented in binary variables.

### 2.3. Estimation

The individual-level fixed effects were controlled using linear probability models for employment and ordinary least squares models for wage.

The fixed effects models are:

(1) $$Y_{i,t}=\beta_{0}+\beta_{1}UW_{i,t-1}+\beta_{2}OWOB_{i,t-1}+\beta_{3}\;Job\;Effort_{i,t-1}\;+\beta_{4}UW_{i,t-1}\times Job\;Effort_{i,t-1}+\beta_{5}OWOB_{i,t-1}\times Job\;Effort_{i,t-1}\;+\beta_{6}X_{i,t}+\mu_{i}+\epsilon_{i,t}$$

The subscripts *i* and *t* in the equation represent individual and time. *Y* indicates status of employment or linear log monthly wages. *UW* and *OWOB* represent the underweight and the overweight and obese, respectively. *Job Effort* represents lagged job hunting or job training. Job hunting is either a linear variable for duration of job hunting in weeks or a dummy indicator for having any experience in job hunting, and job training was measured as a binary variable of either having or not having any experience in training. The β*s* are estimated parameters and *X* represents covariates. μ is individual-level permanent fixed effects and ϵ is an independently identically distributed error term.

This study was reviewed and approved by the Review Board of the Yonsei Institute of Pharmaceutical Sciences (7001988-201704-HR-175-01E).

## 3. Results

Table 1 shows the distributional characteristics of the sample by gender. The current employment rates were 71.36% for women and 70.04% for men. While the employment rates for women were higher, the average monthly wage levels for women were lower, with 1.596 M KRW for women and 1.948 M KRW for men (Table 1).

On average, women tended to have more job-hunting experience and to invest more time in job hunting. In addition, women were reported to have more experience in job training by approximately 10%. The proportion of underweight and of overweight and obese was drastically different between men and women. For instance, only 2.21% of males were reported to be underweight, while 23.96% of females were underweight, and 27.05% of men fell in the overweight and obese category, while only 4.46% of women were either overweight or obese (Table 1).

**Figure 2:**
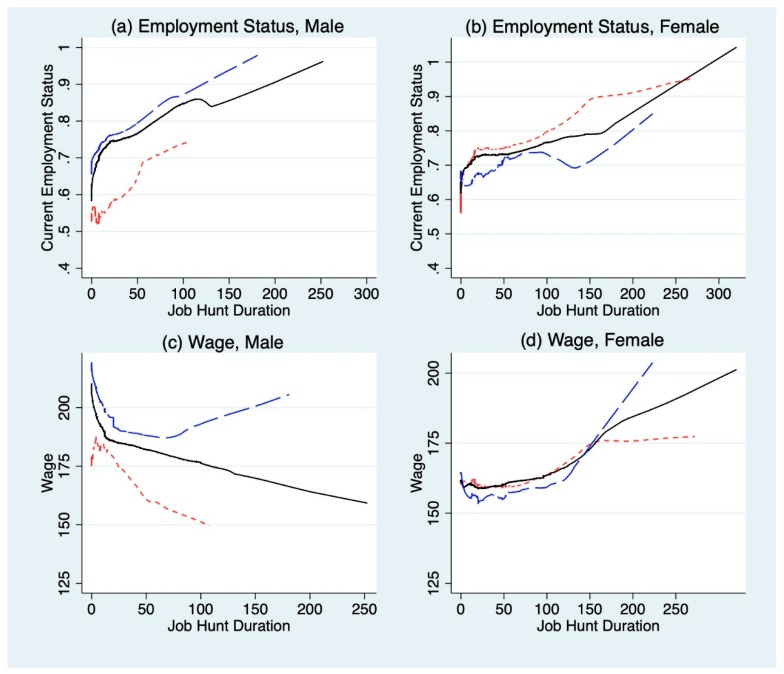
Lowest curves for each dependent variable (current employment status and wage) and job hunt duration by gender and BMI classification group. Note: The X-axis represents job hunt duration, which is measured in weeks. The Y-axis for (**a**,**b**) represents current employment status, while that of (**c**,**d**) represents wage in KRW in units of ten thousand. The solid black lines represent the normal-weight BMI group, the blue dashed lines represent the overweight and obese category, and the red dashed lines represent the underweight individuals.

Figure 2 shows the probability of employment and monthly wage level depending on the respondents’ gender, BMI classification group, job hunting duration, and job training experiences. For all three BMI classification groups, the probability of employment increases as one’s time spent on job hunting increases. While there is no clear distinction between the BMI groups, the magnitude of the increase in probability of employment differs by gender. The effect of job hunting on employment is greatest for overweight and obese men but lowest for that of women. Likewise, the effect of job hunting on employment is lowest for underweight men but highest for underweight women. The same can be observed for wage level. Wage increases for both genders when comparing each of the three BMI classification groups in the order from the underweight category to the overweight and obese BMI group.

**Figure 3:**
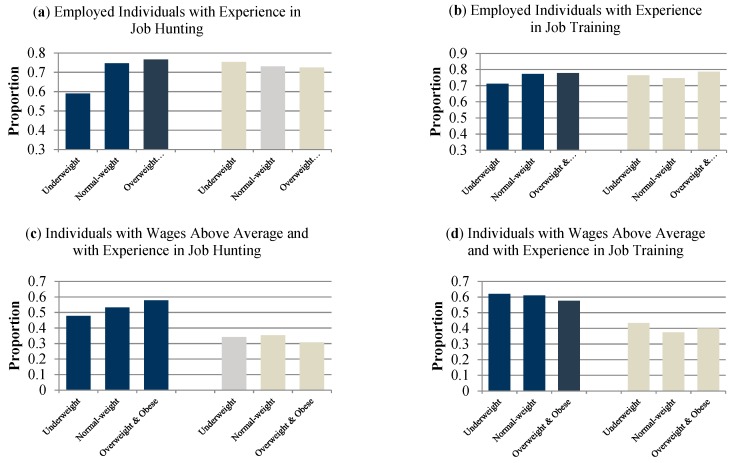
Proportions of employed individuals and of individuals with wages above average with experience in job hunting and of job training by gender and BMI classification groups. Note: The dark blue bars represent men and the beige bars represent women. (**a**,**b**) depicts results regarding the dependent variable of current employment status and (**c**,**d**) analyzes the dependent variable of wage above average.

Figure 3 includes information on the proportions of employed individuals and of individuals with wages above average with experience in job hunting and job training by gender and BMI classification groups. The proportion of underweight employed men is lowest and of overweight and obese men is highest. The opposite is true for employed women. The proportion of individuals with wages above average and with experience in job hunting increases with BMI for men but decreases with BMI for women.

Table 2 shows the results from the fixed-effects models for the association between job efforts and the three dependent variables. Model 1 focuses on the respondents’ number of weeks spent job hunting and on the number of job training experiences they have undergone throughout their lifetime. In Model 1, although they were not statistically significant, the results indicate a positive association between job hunting duration and employment status for both genders. In addition, there is a positive association between number of job training experiences and employment status for both genders, but the regression coefficient for women is noticeably higher. Contrary to that on employment status, in terms of the dependent variable of monthly wages, the positive effect on the number of job training experiences for men is greater than for women and is, unlike that of women, statistically significant (7.0% higher monthly wages per unit increase in the number of job training experiences). Model 2 of Table 2 shows estimation results on the effect of having any experience in job hunting and in job training on labor market outcomes. The results indicate that job hunting benefits women’s employment status more than men’s, with results showing that any job-hunting experience was associated with 18.4% points and 26.6% points times higher likelihood of employment for men and women, respectively. For monthly wages, any job training experience was associated with higher monthly wages by 13.7% for men and 7.4% for women.

Table 3 shows the results from the fixed-effects models for the association between each BMI group and labor market outcomes, regarding employment status and wage level. Compared to their normal-weight counterparts, underweight women were 5.34% points more likely to be employed. In addition, compared to those of their normal-weight counterparts, overweight and obese men experienced higher wages by 6.71% (Table 3).

Table 4 presents the incremental changes in the employment status and monthly wage with respect to BMI classifications by job hunting duration. Compared to that of normal-weight female individuals, underweight women were 4.56 times more likely to be employed at each one-week increase of job-hunting duration. For monthly wage, overweight and obese men had 6.8 times higher wages than their normal-weight counterparts as their job-hunting duration increased by a week. When observing job hunting as a binary variable for any experience versus no experience, underweight women with any job-hunting experience had a higher likelihood of employment (by 5.59 times) than their normal-weight counterparts with such experience (Model 2 of Table 4).

Table 5 depicts associations between labor market outcomes and experience in job training, which has only been analyzed as a binary variable for any experience versus no experience due to insufficient amount of data. Compared to normal-weight men, underweight men are 12.02% points less likely to be employed despite their experience in job training. The extent of rewards for being underweight for women was 6.96% points when job training experience was present, compared to no such experience for women. Overweight and obese men are also reported to have higher monthly wages (by 9.02%) than normal-weight men when they had any job-hunting experience. No such advantage in earnings by job hunting experience was observed for overweight and obese women.

## 4. Discussion

The results from this study demonstrate the contrasting effects job hunting and job training play on one’s employment status and wage level in each BMI classification group. Despite having more direct employment efforts of either or both experience in job hunting and job training, compared to normal-weight men, underweight men were reported to have a disadvantage in both dependent variables, whereas overweight and obese men were shown to have an advantage in wages. Contrastingly, underweight women were observed to have an advantage, while overweight and obese women experienced lower employment rates compared to their normal-weight counterparts.

It is important to note that the lower employment rate for men than for women could be accounted for by the mandatory twenty-one-month-long national military service for South Korean men. Since national military service is usually completed in their early twenties, it is possible that men are more likely to start their careers at an older age than women.

Body weight penalty is increasingly noticed in the labor market, preventing obese applicants from getting favorable jobs and earning comparable wages compared to their normal-weight counterparts [21]. Previous studies of obesity and employment have found that obesity plays a significant role in employment status and wage level. The results from a survey conducted on a nationally representative sample of adults in the United States show that a significant percentage of obese individuals, particularly the young, female, African American, and shorter respondents, have faced body weight penalty in employment [6]. Results showed drastic differences in reported body weight penalty between the very obese and normal-weight categories: 27.1% very obese and 0.7% normal-weight females, and 12.1% very obese and 0.7% normal-weight males had experienced such penalty in the labor market [6].

Direct distaste is present on those who are visibly overweight or obese [11]. Physically obese people are seen to have less desirable traits, causing them to be less liked and viewed less favorably by both the employees and their customers [22]. This discriminatory behavior is present for all age types and is inclined to start at a young age. For instance, a study done by Staffieri (1967) showed that children describe visibly obese children as lazy, dirty, unintelligent, and untrustworthy [23]. Furthermore, the conception that obese people tend to rely more on medical benefits and thus face more hospital costs is a commonly perceived notion in general society, and as a result from that, two major effects take place: a reduction in employment rates, as well as a reduction in occupational prestige for the obese [24,25].

A few studies report gender differences in the relationship of body weight status with labor market outcomes in Korea. Park and Lee (2010) reported a height wage penalty only for men, and Kim and colleagues (2012) reported the obesity wage penalty only for women in their 20s [26,27]. It is also worth noting the potential differences in social norms regarding overweight or obesity by gender; Lee and Sun (2013) reported that women tended to self-classify as obese despite being normal weight, whereas men were likely to classify themselves as non-obese despite being obese [28]. A psychological research also suggested that women tended to be evaluated more than men on their physical appearance, including their physique, which led women to invest more in their appearance to look more satisfactory for the public. Accordingly, when examining the labor market, the employment rate for underweight women is higher [29].

This paper improves on this literature by concentrating on young adults and by assessing any moderation of such penalty by direct employment efforts of job hunting and job training experiences. Assuming that a greater amount of experience in job hunting and in job training produces a greater possibility in employment, an assessment of these two factors provides more insight into individuals’ efforts and resulting statuses in employment. We also measured wages to accurately assess the relationship between obesity and job market performances. Another quality of this study is that the study extracted data from young adults. Recruiters are more susceptible to judging young applicants based on physical appearance because young adults are generally new to the labor market and thus tend to have fewer qualities written down on their resumes [30].

There are limitations of this study that should be addressed. Firstly, It has been reported that BMI fails to take traits like muscle mass and bone density into account [31,32]. However, this system of body fat measurement has been used for this study because the aim was to distinguish the difference in effects of direct employment efforts based on BMI. In situations such as applying for jobs, physical appearance, rather than nonphysical attributes, plays a notable role in the work force. Therefore, the use of BMI is an adequate method for observing the associations between the variables. In addition, the information used to compute BMI for this study, which was height and weight, was self-reported data gathered from surveys. Self-reported height and weight tend to be overestimated and underestimated, respectively. Therefore, it is possible to state that the BMI calculations used for this study may not provide accurate depictions of the study’s results [32,33]. However, some studies discuss that, when analyzing the final results, self-reported numbers and precise numbers do not carry any significant differences [8].

Potential bias due to self-report also exists for other information used in the present study, such as job training. The respondents were required to self-report their answer of either yes or no to the question asking whether the subject had ever done job training for the purpose of employment or job development. The noticeable difference between the number of male and female participants of this study should be addressed. Another limitation to be discussed is the study’s exclusion of juniors. Since KEEP gathered different information from high school and middle school students by sending a different questionnaire to each—one for middle school students focused on school life and one for high school students focused on employment—there was no job-related information that could be obtained from the middle school students. However, since only high school students were evaluated for this study, we were able to get valuable results that show their employment statuses during a similar period of time. The inclusion of only high school students also gives the study an advantage in focusing on the population transferring from academics to employment.

While obesity has been an issue prevalent in many developed countries like the United States and South Korea, a similar rise has also been observed in developing countries in especially Latin America and Asia [34]. These developing countries may even surpass the mean BMI level of developed countries in the near future because of the recent drastic change in diet and exercise from different socioeconomic backgrounds [35]. Furthermore, as obesity rates have been on an incline, there is an evident difference in proportion of presence among genders. A greater portion of females than of males is reported to fall into the obese category [35]. Therefore, studies on the impact of obesity need to be expanded on developing countries.

The current study contributes to building up global evidence on the spillover effect of obesity beyond health, which can be used for initiatives for preventive interventions to contain obesity, particularly for adolescents who move on to early adulthood and enter the job market. Our findings also suggest the importance of public health interventions to manage obesity in Korea. The moderating role of job experience and job training in the relationship of obesity with job performances would vary by society as each society has specific cultural and contextual environments to influence each attribute [36]. 

## 5. Conclusions

Independent of one’s experience in job hunting and job training, weight penalty plays an influential role for both genders of young adults in areas of employment status and wage level.

## Figures and Tables

**Table 1:** Descriptive statistics (whole sample: N = 15,899).

Variables	Mean (Standard Deviation) [Minimum, Maximum]	
	Men (N = 10,065)	Women (N = 5834)
**Dependent Variables**		
Current employment status	15.616 (23.7615) [0,252]	28.1212 (33.5558) [0,320]
Monthly wage ^a^	1948.72 (950.44) [20,40,000]	1596.93 (517.25) [10,6500]
**Key Independent Variables**		
Job hunting duration (weeks) ^b^	15.616 (23.7615) [0,252]	28.1212 (33.5558) [0,320]
Job hunting experience		
No	0.3353 (0.4721)	0.1481 (0.3552)
Yes	0.6647 (0.4721)	0.8519 (0.3552)
Number of job training experiences	0.2778 (0.8262) [0,^18^]	0.3603 (0.7922) [0,^9^]
Job training experience		
No	0.8324 (0.3735)	0.7557 (0.4297)
Yes	0.1676 (0.3735)	0.2443 (0.4297)
BMI (lagged) ^c^	23.5024 (3.1562) [12.6247,39.3853]	20.2417 (2.4167) [14.5243,34.6021]
BMI groups (lagged)		
Underweight ^d^	0.0221 (0.1469)	0.2396 (0.4269)
Normal-weight ^e^	0.7074 (0.4550)	0.7158 (0.4511)
Overweight and Obese ^f^	0.2705 (0.4443)	0.0446 (0.2064)
**Covariates**		
Highest level of educational attainment		
High school diploma or less	0.1942 (0.3956)	0.2113 (0.4083)
Undergraduate diploma or more (reference)	0.5784 (0.4938)	0.7088 (0.4544)
N/A	0.2297 (0.4207)	0.0828 (0.2756)
College Grade ^g^		
A range	0.2630 (0.4403)	0.2873 (0.4525)
B range (reference)	0.4362 (0.4959)	0.4921 (0.5000)
C range	0.1168 (0.3212)	0.1023 (0.3031)
D/F range	0.0237 (0.1523)	0.0141 (0.1177)
N/A	0.4451 (0.4970)	0.3759 (0.4844)
Second language	0.5385 (0.4985)	0.4784 (0.4996)
Father’s average monthly income ^h^		
Under 2M KRW (reference)	0.4077 (0.4914)	0.3356 (0.4722)
2M–3M KRW	0.1238 (0.3294)	0.1215 (0.3268)
3M–5M KRW	0.1124 (0.3158)	0.1090 (0.3117)
Above 5M KRW	0.0496 (0.2171)	0.0406 (0.1974)
N/A	0.3066 (0.4611)	0.3932 (0.4885)
Mother’s average monthly income ^h^		
Under 2M KRW (reference)	0.5499 (0.4975)	0.4553 (0.4980)
2M–3M KRW	0.1193 (0.3242)	0.1260 (0.3319)
3M–5M KRW	0.0349 (0.1835)	0.0370 (0.1888)
Above 5M KRW	0.0263 (0.1601)	0.0213 (0.1442)
N/A	0.2695 (0.4437)	0.3605 (0.4802)
Self-reported health status		
Healthy	0.6205 (0.4853)	0.4633 (0.4987)
Average (reference)	0.3216 (0.4671)	0.4152 (0.4928)
Unhealthy	0.0579 (0.2336)	0.1215 (0.3268)
Average hours of sleep per day	6.6439 (1.0922) [3,20]	6.8426 (1.1526) [3,12]
Breakfast		
Often	0.3058 (0.4608)	0.2952 (0.4562)
Sometimes (reference)	0.3830 (0.4861)	0.3915 (0.4881)
Never	0.3112 (0.4630)	0.3133 (0.4639)
Perceived discrimination from appearance	0.0609 (0.2392)	0.0902 (0.2864)
Perceived awareness of self		
Aptitude	0.9408 (0.2360)	0.9069 (0.2906)
Interests	0.9391 (0.2392)	0.9244 (0.2644)
Value	0.9634 (0.1877)	0.9491 (0.2198)
Decisiveness	0.9294 (0.2562)	0.8809 (0.3240)
Perseverance	0.9332 (0.2496)	0.9042 (0.2944)
Image/Reputation	0.9663 (0.1804)	0.9607 (0.1942)

^a^ Measured in 1000 KRW, where 1000 KRW is roughly 0.82 USD (N = 6186 for men; N = 3710 for women). ^b^ Total number of weeks spent on job-hunting. ^c^ BMI from the previous wave. ^d^ “Underweight” classified as BMI < 18.5. ^e^ “Normal-weight” classified as 18.5 ≤ BMI ≤ 24.9. ^f^ “Overweight and Obese” classified as BMI > 24.9. ^g^ Grades converted to a single, unified system of letter grades. ^h^ Measured in 1 million (M) KRW, where 1000 KRW is roughly 0.82 USD.

**Table 2:** Marginal effect of job efforts on labor market outcomes: individual-level fixed-effects models.

Key Independent Variable	Regression Coefficient (Standard Error)			
	Employment		Ln (Monthly Wage)	
	Men	Women	Men	Women
	(N = 10,065)	(N = 5834)	(N = 6186)	(N = 3710)
**Model 1**				
Job hunting duration (weeks)	0.0007 (0.0003)	0.0005 (0.0002)	0.0006 (0.0004)	0.0004 (0.0003)
Number of job training experiences	0.0017 (0.0094)	0.0193 (0.0155)	0.0704 *** (0.0121)	0.0142 (0.0142)
**Model 2**				
Any job hunting experience ^a^	0.1844 *** (0.0165)	0.2663 *** (0.0328)	0.1594 *** (0.0271)	0.0572 (0.0390)
Any job training experience ^a^	0.0090 (0.0216)	0.0676 (0.0322)	0.1377 *** (0.0286)	0.0744 ** (0.0309)

** *p*-value < 0.05, *** *p*-value < 0.01. ^a^ Dummy indicator.

**Table 3:** Association between Y and BMI classification groups (%).

Key Independent Variable	Regression Coefficient			
	(Standard Error)			
	Employment		Ln (Monthly Wages)	
	Men	Women	Men	Women
	(N = 10,065)	(N = 5834)	(N = 6186)	(N = 3710)
**BMI Group**				
Underweight (lagged)	−0.0510 (−0.0412)	0.0534 ** (−0.0215)	−0.0222 (−0.0492)	−0.0148 (−0.0204)
Overweight and Obese (lagged)	−0.0102 (−0.0154)	−0.0120 (−0.0371)	0.0671 *** (−0.0186)	0.03556 (−0.0354)

** *p*-value < 0.05, *** *p*-value < 0.01.

**Table 4:** Changes in the incremental effect of the BMI group on labor market outcomes by job hunting or training efforts.

Key Independent Variable	Regression Coefficient (Standard Error)			
	Employment		Ln (Monthly Wage)	
	Men	Women	Men	Women
	(N = 10,065)	(N = 5834)	(N = 6186)	(N = 3710)
**Model 1**				
Job hunting duration (weeks) × Underweight (lagged)	−3.2445 (−4.4559)	4.5600 * (−2.5537)	0.4016 (−5.4427)	−2.5254 (−2.3993)
Job hunting duration (weeks) × Overweight and Obese (lagged)	−0.0096 (−1.7328)	−4.7809 (−4.5080)	6.7991 *** (−2.0866)	3.2500 (−4.3516)
**Model 2**				
Any Experience of job hunting ^a^ × Underweight (lagged)	−7.8786 (−4.9282)	5.5918 ** (−2.2790)	0.54088 (−5.7674)	−1.9525 (−2.1290)
Any Experience of job hunting ^a^ × Overweight and Obese (lagged)	−1.5327 (−1.7238)	−2.8070 (−3.9089)	8.2943 *** (−2.0755)	4.4907 (−3.7821)

^a^ Dummy indicator. * *p*-value < 0.1, ** *p*-value < 0.05, *** *p*-value < 0.01.

**Table 5:** Changes in the incremental effect of BMI group on labor market outcomes by job training efforts.

Key Independent Variable	Regression Coefficient (Standard Error)	
	Men	Women
	(N = 10,065)	(N = 5834)
**Model 1:**	**Employment**	
Any Experience of job training ^a^ × Underweight (lagged)	−0.1202 ** (0.0524)	0.0696 ** (0.0280)
Any Experience of job training ^a^ × Overweight and Obese (lagged)	−0.0639 (0.0176)	0.0222 (0.0556)
**Model 1:**	**Ln (monthly wage)**	
Any Experience of job training ^a^ × Underweight (lagged)	−0.0055 (0.0571)	−0.0093 (0.0249)
Any Experience of job training ^a^ × Overweight and Obese (lagged)	0.0902 *** (0.0204)	0.0609 (0.0523)

^a^ Dummy indicator. ** *p*-value < 0.05, *** *p*-value < 0.01.

## References

[1] Hales C.M., Carroll M.D., Fryar C.D., Ogden C.L. (2017). Prevalence of Obesity Among Adults and Youth: United States, 2015–2016.

[2] Johnson D. (2018). The obesity epidemic. Integr. Stud..

[3] Devaux M.G.S., Goryakin Y., Cecchini M., Huber H., Colombo F. (2017). OECD Obesity Update 2017.

[4] Greve J. (2008). Obesity and labor market outcomes in Denmark. Econ. Hum. Biol..

[5] Lindeboom M., Lundborg P., van der Klaauw B. (2010). Assessing the impact of obesity on labor market outcomes. Econ. Hum. Biol..

[6] Roehling M.V., Roehling P.V., Pichler S. (2007). The relationship between body weight and perceived weight-related employment discrimination: The role of sex and race. J. Vocat. Behav..

[7] Wing R.R., Greeno C.G. (1994). 9 Behavioural and psychosocial aspects of obesity and its treatment. Bailliere’s Clin. Endocrinol. Metab..

[8] Cawley J. (2004). The impact of obesity on wages. J. Hum. Resour..

[9] Vaidya V. (2006). Psychosocial aspects of obesity. Health and Treatment Strategies in Obesity.

[10] Hammond R.A., Levine R. (2010). The economic impact of obesity in the United States. Diabetes Metab. Syndr. Obes. Targets Ther..

[11] Baum C.L., Ford W.F. (2004). The wage effects of obesity: A longitudinal study. Health Econ..

[12] Malnick S.D., Knobler H. (2006). The medical complications of obesity. J. Assoc. Physicians.

[13] Wyatt S.B., Winters K.P., Dubbert P.M. (2006). Overweight and obesity: Prevalence, consequences, and causes of a growing public health problem. Am. J. Med Sci..

[14] Reilly J.J., Methven E., McDowell Z.C., Hacking B., Alexander D., Stewart L., Kelnar C.J. (2003). Health consequences of obesity. Arch. Dis. Child..

[15] Bhattacharya J., Bundorf M.K. (2009). The incidence of the healthcare costs of obesity. J. Health Econ..

[16] Morris S. (2007). The impact of obesity on employment. Labour Econ..

[17] Caliendo M., Lee W.S. (2013). Fat chance! Obesity and the transition from unemployment to employment. Econ. Hum. Biol..

[18] Mincer J. (1988). Job Training, Wage Growth, and Labor Turnover.

[19] Bartel A.P. (1995). Training, wage growth, and job performance: Evidence from a company database. J. Labor Econ..

[20] Krueger A., Rouse C. (1994). New Evidence on Workplace Education.

[21] Cawley J., Danziger S. (2004). Obesity as a Barrier to the Transition from Welfare to Work.

[22] Vartanian L. (2010). Obese people vs. Fat people: Impact of group label on weight bias. Eat. Weight Disord. Stud. Anorex. Bulim. Obes..

[23] Staffieri J.R. (1967). A study of social stereotype of body image in children. J. Personal. Soc. Psychol..

[24] Avsar G., Ham R., Tannous W.K. (2017). Modelling gender differences in the economic and social influences of obesity in Australian young people. Int. J. Environ. Res. Public Health.

[25] Averett S.L. (2011). Labor market consequences: Employment, wages, disability, and absenteeism. The Oxford Handbook of the Social Science of Obesity.

[26] Kim Y., Park G. (2012). Effects of height and obesity on wage: Gender differences. Sol. Sci. Stud..

[27] Park K., Lee I. (2010). Height premium in Korean labor market. Korean J. Labor Econ..

[28] Lee Y., Sun L. (2013). The study of perception in body somatotype and dietary behaviors-the comparative study between Korean and Chinese college students. Korean J. Commun. Nutr..

[29] Muth J.L., Cash T.F. (1997). Body-image attitudes: What difference does gender make?. J. Appl. Soc. Psychol..

[30] Caliendo M., Gehrsitz M. (2016). Obesity and the labor market: A fresh look at the weight penalty. Econ. Hum. Biol..

[31] Rothman K.J. (2008). BMI-related errors in the measurement of obesity. Int. J. Obes..

[32] Burkhauser R.V., Cawley J. (2008). Beyond BMI: The value of more accurate measures of fatness and obesity in social science research. J. Health Econ..

[33] Gosse M. (2014). How accurate is self-reported BMI?. Nutr. Bull..

[34] Popkin B.M. (1998). The nutrition transition and its health implications in lower-income countries. Public Health Nutr..

[35] Bhurosy T., Jeewon R. (2014). Overweight and obesity epidemic in developing countries: A problem with diet, physical activity, or socioeconomic status?. Sci. World J..

[36] Kim T.H., Han E. (2015). Impact of body mass on job quality. Econ. Hum. Biol..

